# Morphological and molecular evidence support the intertidal barnacle *Octomeris intermedia* Nilsson-Cantell, 1921 (Thoracica, Chthamalidae) as a valid species in Indo-Pacific waters

**DOI:** 10.3897/zookeys.914.49328

**Published:** 2020-02-20

**Authors:** Benny K.K. Chan, Yao Feng Tsao, Monthon Ganmanee

**Affiliations:** 1 Biodiversity Research Center, Academia Sinica, Taipei 115, Taiwan Biodiversity Research Center, Academia Sinica Taipei Taiwan; 2 Faculty of Agricultural Technology, King Mongkut's Institute of Technology Ladkrabang, Chalongkrung Road, Ladkrabang, Bangkok 10520, Thailand King Mongkut's Institute of Technology Bangkok Thailand

**Keywords:** Barnacles, biogeography, molecular taxonomy

## Abstract

*Octomeris* is a chthamalid intertidal barnacle with eight shell plates. There are currently two species of such barnacles: *O. brunnea* Darwin, 1854 (type locality in the Philippines), common in the Indo-Pacific region, and *O. angulosa* Sowerby, 1825, only recorded in South Africa. *Octomeris intermedia* Nilsson-Cantell, 1921, identified from the Mergui Archipelago in Myanmar, was considered to be conspecific with *O. brunnea* by Hiro (1939) based on samples collected in Taiwan. The morphological differences in shell and opercular plates between *O. brunnea* and *O. intermedia* are believed to be intra-specific variations due to different degrees of shell erosion. In the present study, the genetic and morphological differentiations of *Octomeris* in the Indo-Pacific region were examined. This study found two molecular clades (with inter-specific differences) based on the divergence in the COI genes, and the species also have distinct geographical distributions. The *Octomeris brunnea* clade covers samples collected from the Philippines and Taiwan waters and the other clade, which we argue is *O. intermedia*, is distributed in Phuket and Krabi, Thailand and Langkawi, Malaysia. Phuket and Krabi are located approximately 300 km south of the Mergui Archipelago, the type locality of *O. intermedia*. The morphology of samples collected from Thailand fits the type description of *O. intermedia* in Nilsson-Cantell (1921). Our study concludes that *O. intermedia* is a valid species based on morphological and molecular evidence.

## Introduction

*Octomeris* is a chthamalid intertidal barnacle with eight shell plates, in contrast to most of chthamalids which have four or six shell plates. In the early 19^th^ century, studies on the biology and ecology of *Octomeris* were very rare because this species inhabits shaded habitats and its presence was often overlooked (Nilsson-Cantell 1938). A recent molecular phylogenetic analysis of the family Chthamalidae included two species of *Octomeris* (Pérez-Losada et al. 2012) and considered *Octomeris* as paraphyletic; molecular evidence does not support the hypothesis that plate number decreased from eight plates to six, then four in the chthamalid evolution (Pérez -Losada et al. 2012).

**Figure 1.**
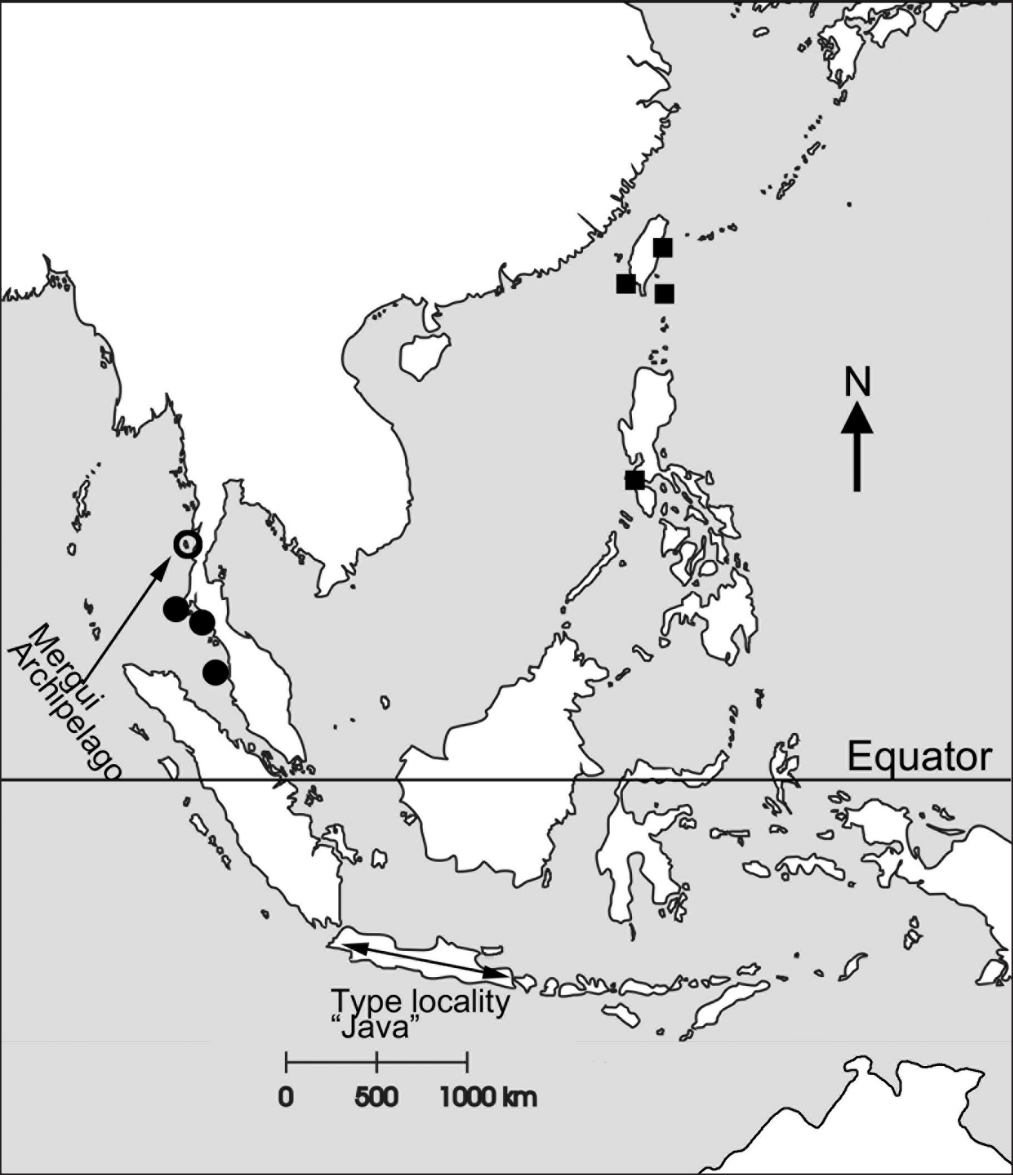
Sampling locations for *Octomeris intermedia* (black circles) and *Octomeris brunnea* (black squares). Open circle indicates the sampling location of *O. intermedia* in the Mergui Archipelago stated in Nilsson-Cantell (1938).

In the Indo-Pacific region, *Octomeris* was considered to be composed of four species: *O. brunnea*, *O. angulosa*, *O. sulcata*, and *O. intermedia*. *Octomeris sulcata* has a strongly fused scutum and tergum, and Poltarukha (1996) relocated *O. sulcata* to the monotypic genus *Pseudoctomeris*. Chan et al. (2017) repositioned *Pseudoctomeris* from Chthamalidae to Pachylasmatidae based on multiple marker molecular analyses, leaving three species in *Octomeris*. *Octomeris brunnea* was described by Darwin (1854) from the Philippine archipelago; it has a brown colored depressed shell and longitudinal furrows on its surface. The tergal and scutal margins of the opercular plates are straight. *Octomeris angulosa* was described by Sowerby (1825) from the Cape of Good Hope in South Africa as having a dirty white strong conical shell and coarsely crenated shell plates (see re-description in Darwin 1854). *Octomeris angulosa* is common in wave-exposed shores in South African waters and often interacts with *Tetraclita serrata* (Boland 1997). *Octomeris intermedia* was described by Nilsson-Cantell (1921) from Java (note the erratum on the type locality (South Atlantic Ocean in Nilsson-Cantell, 1921) stated in Nilsson-Cantell (1937); Fig. 1), having a depressed shell and sinuous tergal and scutal margin. Nilsson-Cantell (1938) further recorded *O. intermedia* in the Mergui Archipelago in the Malay Peninsula (Fig. 1). Hiro (1939), however, collected a different size range of *O. brunnea* in Taiwan and observed that there is great variation in the shape of scutum and tergum at different ages and with different degrees of erosion. Highly eroded large individuals have a smooth flattened shell and a sinuous tergal and scutal margin, which resemble the morphology of *O. intermedia*. Juvenile and uneroded specimens represent the morphology of *O. brunnea*, which has longitudinally furrowed shells and straight scutum and tergum junction. Hiro (1939) concluded that *O. intermedia* and *O. brunnea* are conspecific. The conclusion of Hiro (1939) was further supported by Pope (1965), who examined *O. brunnea* in Australian waters and suggested that *O. intermedia* is an older specimen of *O. brunnea*. However, Hiro (1939) did not include *O. intermedia* in the geographical range suggested by Nilsson-Cantell (1921, 1938) (Java and the Mergui Archipelago), nor did the former compare the latter's samples of *O. brunnea* collected from Taiwan. To further test the conclusion by Hiro (1939), a combined morphological and molecular approach, known as integrative taxonomy (Dayrat 2005). is needed to compare *O. intermedia* collected from Java, Mergui Archipelago, and their adjacent waters with *O. brunnea* and ascertain the taxonomic status of these two species. In the present study, we collected *O. intermedia* from Phuket and Krabi, Thailand (300 km south of the Mergui Archipelago) and Langkawi, Malaysia, and *O. brunnea* from Taiwan and the Philippines; these samples cover different sizes and degrees of erosion. The mitochondrial cytochrome C oxidase subunit I (COI) and 12S rRNA genes were used as genetic markers to test the hypothesis that *O. intermedia* is an eroded form of *O. brunnea* in the Indo-Pacific region.

## Materials and methods

### Study sites and sample collections

**Figure 2.**
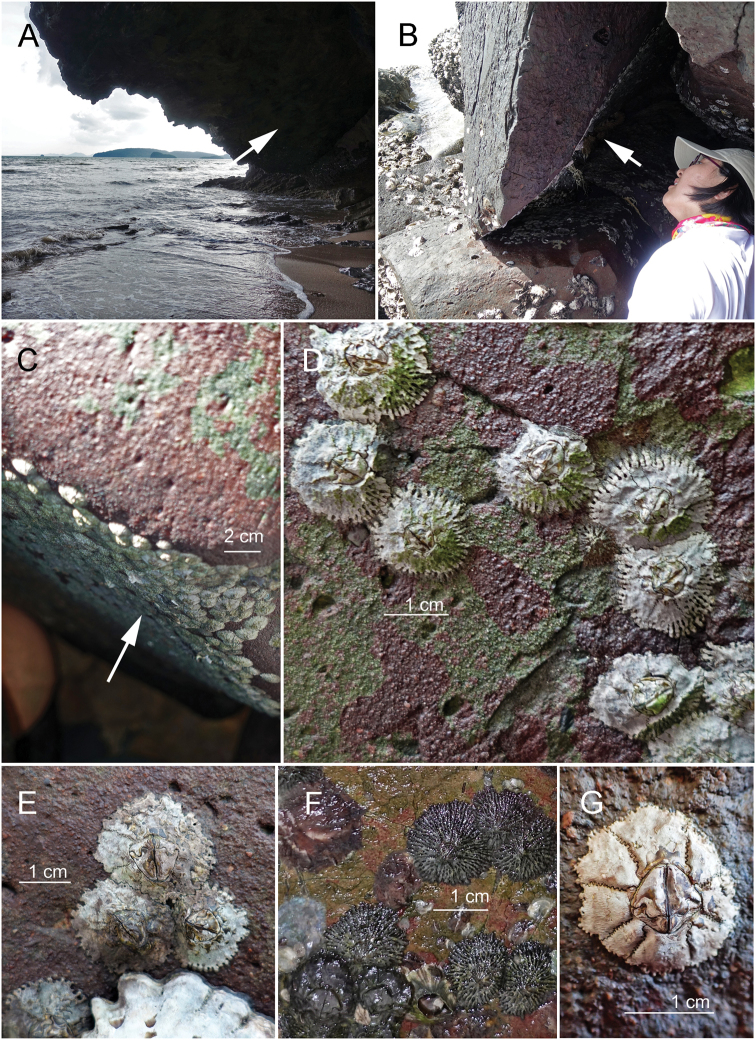
**A** Krabi, Thailand, showing *O. intermedia* at the overhang of an intertidal cave (indicated by white arrow). **B** Hey or Coral Island, Phuket, showing that *O. intermedia* occur on shaded rocks. **C***O. intermedia* can occupy up to 100% cover under a shaded rock in Thailand. **D***Octomeris intermedia*, showing partially eroded samples, with longitudinal furrows at the base of the shell plates. **E***O. intermedia*, eroded samples, showing shell with a smooth surface. **F***O. intermedia*, uneroded samples, showing longitudinal furrows on the surface. **G***O. brunnea* on Lanyu, Taiwan. Shell is more conical than *O. intermedia*. Eroded sample with smooth shell plates.

Samples of *Octomeris intermedia* were collected from Hey (or Coral) Island, Phuket (7°44'47"N; 98°22'44E) and Ao Nang Beach, Krabi (8°02'08"N; 98°48'57E), Thailand and Langkawi, Malaysia (Fig. 1). *Octomeris* in Thailand inhabits shaded rocks in the high intertidal zone, especially on vertical rock surfaces or shaded overhang surfaces in intertidal sea caves (Figs 1, 2). They can reach a percentage cover of 100% in some of the shaded rocks (Fig. 2C). Samples of *O. brunnea* (Fig. 2G) were collected in Kenting, Green Island, and Lanyu Island in Taiwan and Puerto Galera in the Philippines (Fig. 1). *Octomeris brunnea* was found on shaded rock surfaces and, occasionally, sun-exposed rocks. The abundance of *O. brunnea* was not high, with only a few individuals colonizing a shaded area. Representative specimens were deposited in the Biodiversity Research Museum, Academia Sinica (ASIZCR) and Collections in the first author's laboratory (CEL).

### Morphological analysis

Before dissection, the shape of the junction line of scutum and tergum was recorded for all specimens with different size ranges. The morphological characters of shell parts (wall plates, scutum, and tergum) and somatic bodies (six pairs of cirri, penis, and trophi) were examined. The shells and opercular plates (scuta and terga) were immersed in 20% bleach for ca. 20 minutes to completely dissolve organic tissues, rinsed by purified water for 5 minutes, and air-dried. The cirri, penis, and trophi were dissected, mounted on glass slides, and observed under a light microscope (Olympus BX60, Japan). The morphology of the setae was described following the terminology of Chan et al. (2008).

### DNA extraction, amplification, and sequencing

Total genomic DNA was extracted from the muscle tissue using Qiagen DNeasy® Blood & Tissue Kits (Qiagen, California, USA) according to the manufacturer's instructions. Partial sequences of mitochondrial DNA markers, COI, and 12S rRNA were amplified to reconstruct phylogenetic relationships. The primers used to amplify the sequences in the polymerase chain reaction (PCR) were LCO1490 and HC02198 for COI (Folmer et al. 1994) and 12S-F1 and 12S-R1 for 12S rRNA (Mokady et al. 1994). The PCR solution contained approximately 100–200 ng of template DNA, 0.4 μL each of 10 μM primer, 4 μL of Fast-Run^TM^*Taq* Master Mix with Dye (Protech Technology Enterprise, Taipei, Taiwan), and ddH_2_O to the final volume of 20 μL. PCR reactions were conducted in a DNA Engine Thermal Cycler (Bio-Rad, Richmond, California, USA). The thermal cycle began with an initial denaturation at 94°C for 4 min, then 35 cycles of denaturation at 94°C for 1 min, annealing at 49–51°C for 30 sec, and an extension at 72°C for 1 min (COI) and 30 sec (12S rRNA). The final extension step was at 72°C for 10 min. PCR products were checked by electrophoresis on 1.5 % agarose gel in 1 × TAE buffer. DNA purification and Sanger DNA sequencing were performed by Genomics BioSci & Tech Ltd. (New Taipei City, Taiwan). The sequences were assembled and edited in Geneious 7.0.6 (https://www.geneious.com).

### Phylogeny reconstruction and genetic distances

The phylogenetic trees were reconstructed from COI and 12S rRNA sequences using neighbor-joining (NJ), Bayesian inferences (BI), and maximum likelihood (ML) conducted in MEGA X 10.0.5, MrBayes 3.2.6, and W-IQ-TREE, respectively (Kumar et al. 2018; Nguyen et al. 2015; Ronquist and Huelsenbeck 2003; Trifinopoulos et al. 2016). Selected sequences of Chthamalidae downloaded from GenBank were included in the analysis, and the *Catomerus polymerus* sequence was used as the outgroup (Chan et al. 2018; Chen et al. 2019; Fisher et al. 2004; Pérez-Losada et al. 2004; Pérez-Losada et al. 2012; Wares 2013; Wares et al. 2009) (Table 1).

**Table 1.** Specimen information and GenBank accession numbers for DNA sequences used in this study.

Species	Specimen voucher	Locality	COI	12S	Reference
*Octomeris brunnea*	KT_131_02	Haikou, Pingtung, Taiwan	MN928617	MN928665	This study
	LAN_178_01	Lanyu, Taiwan	MN928618	MN928668	
	LAN_178_02		MN928619	MN928669	
	Octm_b_02	Haikou, Pingtung, Taiwan	MN928620	MN928670	
	Octm_b_03		MN928621	MN928671	
	Octm_G05_01	Puerto Galera, Mindoro, Philippines	MN928622	MN928672	
	Octm_G05_03		MN928623	MN928673	
	Octm_G05_04		MN928624	MN928674	
	Octm_G05_05		MN928625	MN928675	
	Octm_G23_01		MN928626	MN928676	
	Octm_GI_01	Green Island, Taiwan	–	MN928677	
	Octm_GI_02		–	MN928678	
	Octm_GI_03		–	MN928679	
	Octm_sp_01	Shihtiping, Hualien, Taiwan	MN928627	MN928684	
	Octm_TW_02	Haikou, Pingtung, Taiwan	MN928628	MN928685	
	Octm_TW_05		MN928629	MN928686	
	Octm_TW_06		MN928630	MN928687	
	Octm_TW_07		MN928631	MN928688	
	Octm_TW_08		MN928632	MN928689	
	Octm_TW_09		MN928633	MN928690	
	Octm_TW_10		MN928634	MN928691	
	Octm_TW_11		MN928635	MN928692	
*Octomeris intermedia*	CEL_Thai_243_01	Hey Island, Phuket, Thailand	MN928636	MN928655	
	CEL_Thai_243_02		MN928637	MN928656	
	CEL_Thai_243_03		MN928638	MN928657	
	CEL_Thai_243_04		MN928639	MN928658	
	CEL_Thai_243_05		MN928640	MN928659	
	CEL_Thai_243_06		MN928641	MN928660	
	CEL_Thai_243_07		MN928642	MN928661	
	CEL_Thai_243_08		MN928643	MN928662	
	CEL_Thai_243_09		MN928644	MN928663	
	CEL_Thai_243_10		MN928645	MN928664	
	Octm_MA_01	Langkawi, Malaysia	MN928646	MN928680	
	Octm_MA_02		MN928647	MN928681	
	Octm_MA_03		MN928648	MN928682	
	Octm_MA_04		MN928649	MN928683	
	Thai_359_03	Ao Nang Beach, Krabi, Thailand	MN928650	MN928693	
	Thai_359_05		MN928651	MN928694	
	Thai_359_06		MN928652	MN928695	
Octomeris intermedia*		Phuket, Thailand	AY430812	–	Fisher et al. 2004
			–	JX083940	Pérez-Losada et al. 2012
*Catomerus polymerus*		Coledale Beach, Wollogong, Australia	MH791045	MH791045	Chan et al. 2018
*Chamaesipho columna*		Devenport, New Zealand	JX083866	JX083937	Pérez-Losada et al. 2012
*Chamaesipho tasmanica*		Tasmania, Australia	JX083867	–	
			–	AY520681	Pérez-Losada et al. 2004
*Chthamalus challengeri*		Jiangsu, China	KY865097	KY865097	Chen et al. 2019
*Hexechamaesipho pilsbryi* 2	LAN_173_01	Lanyu, Taiwan	MN928653	MN928666	This study
*Hexechamaesipho pilsbryi* 1	LAN_173_03		MN928654	MN928667	
*Microeuraphia rhizophorae*		Panama	FJ845866	–	Wares et al. 2009
*Microeuraphia rhizophorae*		Brazil	–	JX083950	Pérez-Losada et al. 2012
*Nesochthamalus intertextus*		Japan	JX083869	JX083942	
*Notochthamalus scabrosus*		Arica, Chile	NC_022716	NC_022716	Wares 2013
*Octomeris angulosa*		Cape Town, South Africa	AY428049	–	Fisher et al. 2004
*Octomeris angulosa*		Sydney, Australia	–	JX083939	Pérez-Losada et al. 2012
*Pseudoctomeris sulcata*		Japan	JX083865	JX083936	

* The sequences of *O. intermedia* from Fisher et al. (2004) and Pérez-Losada et al. (2012) were designated as *O. brunnea* in their studies.

All the sequences were aligned with ClustalW implemented in Geneious 7.0.6 (https://www.geneious.com). Neighbor-joining trees were generated on the analysis of Kimura 2-parameter (K2P) distances with bootstrap values estimated from 1,000 pseudoreplicates for two markers, separately (Felsenstein 1985; Kimura 1980; Saitou and Nei 1987). Bayesian inferences were conducted with 2 × 10^6^ generations of the MCMC chain. Trees were saved every 1000 generations, and the first 500,000 trees (25%) were discarded as burn-in. Maximum likelihood was conducted with 1,000 bootstrap replicates for a Shimodaira-Hasegawa approximate likelihood ratio test (SH-aLRT) and ultrafast bootstrap approximation (UFB) (Guindon et al. 2010; Hoang et al. 2017). GTR+F+I+G4 and TVM+F+G4 were selected as the best-fit model under the Bayesian information criterion for COI and 12S rRNA, respectively (Kalyaanamoorthy et al. 2017). Genetic distances (K2P) between and within species were calculated by MEGA X 10.0.5 (Kumar et al. 2018).

## Results

### Systematics

**Family Chthamalidae**

**Subfamily Notochthamalinae**

**Genus Octomeris Sowerby 1825**

#### Octomeris brunnea

Taxon classificationAnimaliaSessiliaChthamalidae

Darwin, 1854

22EBAAB5-3E73-5B62-BFB7-7FECE103B54A

Figures 2G , 3 , 4 , 5 , 6 , 7 , 8 , 9 , 15D–F , 16D–F

**Figure 3.**
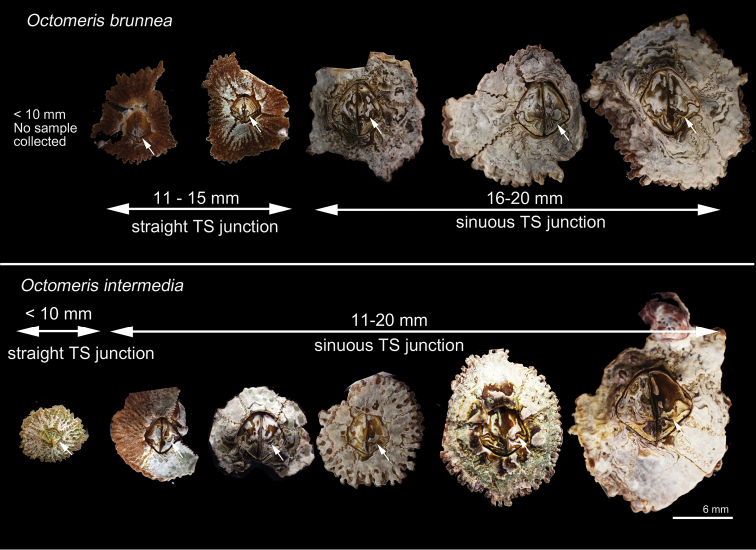
Shape of the tergo-scutal junction (TS junction, indicated by arrows) of *Octomeris brunnea* (CEL-KT-131, Hai Kou, Taiwan) and *O. intermedia* (CEL-Thai-359, Krabi, Thailand) All specimens share the same scale bar.

Octomeris brunnea Darwin, 1854: 484, pl 20, figs 3a, b; Weltner 1897: 274; Gruvel 1905: 197, fig. 217; Nilsson-Cantell 1921: 299, figs 58, 59, pl 3, fig. 7; −1931: 108; −1932, 14; Hiro 1939: 252, figs 3–4, 6a, b; Utinomi 1949: 25; −1958: 307; Endean, Kenny and Stephenson 1956: 122, 127, tab. 1; Endean, Stephenson and Kenny 1956: 332, 336, app II; Pope 1965: 20, figs 1c, 2b, pl 1: figs 3, 6; Newman and Ross 1976: 40; Poltarukha 1996: 992; Liu and Ren 2007: 283, fig. 123; Chan et al. 2009: 153: figs 128–130; Jones 2012: tabs 1, 2.

##### Materials examined.

ASIZCR-000431. Intertidal rocks at General Rock, Green Island, Taiwan (22°40.35'N, 121°29.45E, 16 August 2019, 1 specimen). CEL-Octm_GI_01. Intertidal rocks at General Rock, Green Island, Taiwan (22°40.35'N, 121°29.45'E, 16 August 2019, 5 specimens). CEL-KT-131. Intertidal rocks at Hai Kou, Kenting, Taiwan (22°06.06'N, 120°42.56'E, 4 Dec 2007, 7 specimens). CEL-LAN-178. Intertidal rocks at southern Lanyu, Lanyu, Taiwan (22°00.82'N, 121°33.94E, 19 June 2019, 2 specimens). CEL-Octm_sp_01. Intertidal rocks at Shi-Ti-Ping, Hualien, Taiwan (23°28.56'N, 121°30.41E, 13 May 2009, 1 specimen). CEL-Octm-G05. Intertidal rocks at Puerto Galera, Philippines (02 June 2009, 20 specimens). CEL-Octm-G23. Intertidal rocks at Varadaro Point, Puerto Galrea, Philippines (02 June 2009, 1 specimen).

##### Diagnosis.

Shell eight plated, conically depressed. Shell brown, surface with longitudinal furrows and tergo-scutal junction straight in young and uneroded specimens. Shell gray, surface smooth and tergo-scutal junction sinuous in old and eroded specimens. Maxillule with deep notch at upper 1/3 of cutting edge, lower 1/3 strongly protruded, cutting edge clearly divided into upper, middle, and lower region by the clear notch and protrusion of lower margin.

##### Description.

**Figure 4.**
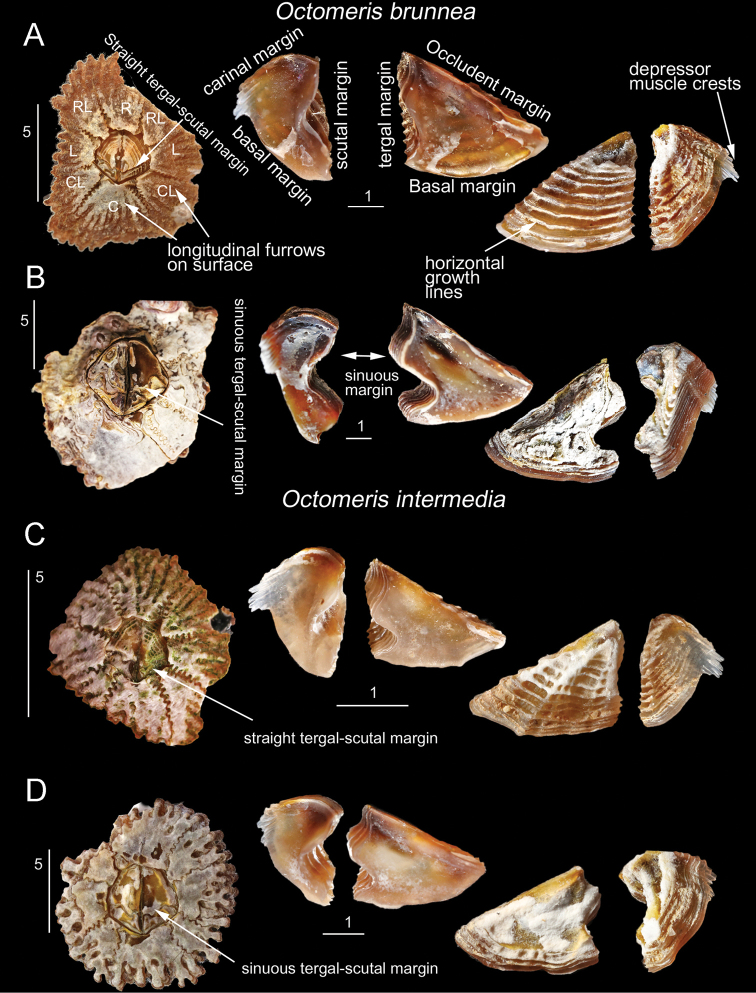
*Octomeris brunnea* (CEL-KT-131, Hai Kou, Taiwan) and *O. intermedia* (CEL-Thai-359, Krabi, Thailand). **A***Octomeris brunnea*, young specimens showing the straight tergo-scutal junction and the inner and outer sides of left scutum and tergum. **B***O. brunnea*, older eroded specimen, showing the sinuous tergo-scutal junction and inner and outer sides of left scutum and tergum. **C***O. intermedia*. Very small individual (shell length < 10 mm) showing the straight tergo-scutal margin and inner and outer sides of scutum and tergum. Note only very small individuals of *O. intermedia* have straight tergo-scutal margin. **D***O. intermedia*. Larger specimens, showing the sinuous tergo-scutal margin and inner and outer surfaces of scutum and tergum. Scale bars in mm.

Shell 8 plated, composed of piece of rostrum (R), carina (C), paired rostro-lateral (RL), carino-lateral (CL) and lateral (L) (Figs 2G, 4A, B). Shell conically depressed. Shell brown, surface with longitudinal furrows and tergo-scutal junction straight in young and uneroded specimens (Figs 3, 4A). Shell grey, smooth, tergo-scutal junction sinuous in old and eroded specimens (Figs 3, 4B). Sutures of shell plates serrated (Figs 2G, 4A, B). In young and uneroded specimens, scutum triangular, outer surface with horizontal growth lines (Fig. 4A). Scutum inner surface brown, tergal and occludent margins straight in young specimens, basal margin slightly convex (Fig. 4A). Tergal margin straight, with conspicuous articular ridge. Adductor muscle scar shallow (Fig. 4A). In older and eroded specimens, tergal margin of scutum strongly sinuous, adductor muscle scar deep (Fig. 4B). Tergum with basal margin strongly bended in an angle, scutal margin straight with deep articular ridge in young specimens, depressor muscle distinct, muscle crests prominent and extended slightly out of the carinal margin of tergum (Fig. 4A). In older and eroded specimens, the scutal margin strongly concaved (Fig. 4B).

**Figure 5.**
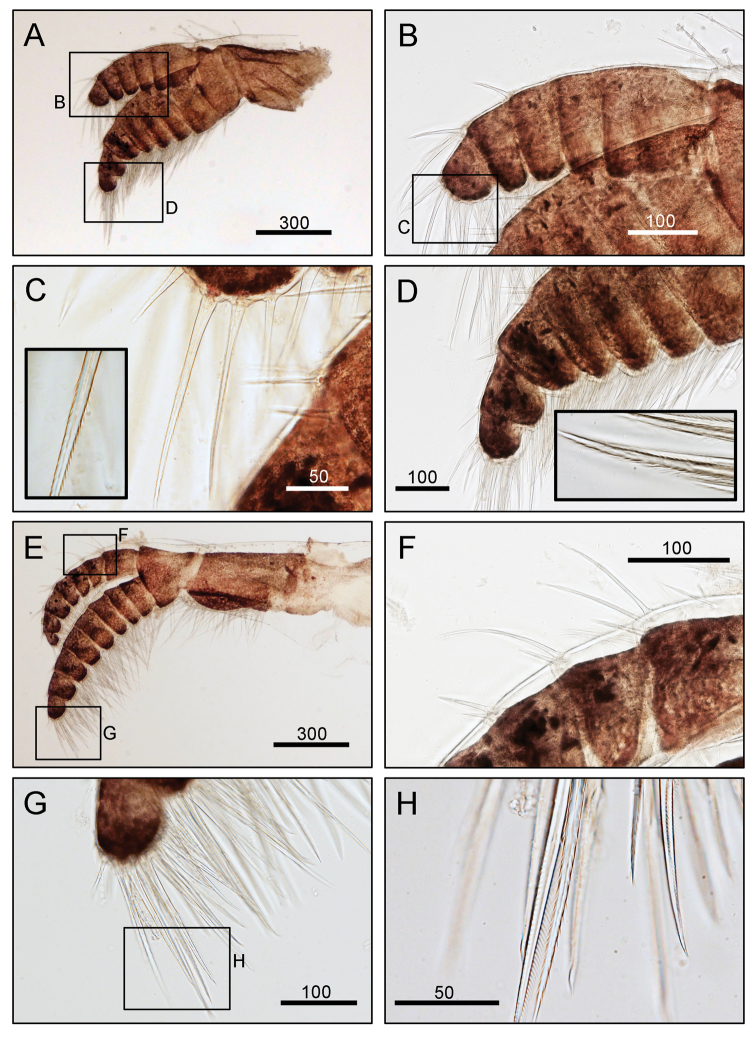
*Octomeris brunnea* (CEL-KT-131, Hai Kou, Taiwan). **A** Cirrus I. **B** Posterior ramus of cirrus I. **C** Bidentate serrate setae at tip of segment. **D** Bidentate serrate setae at tip of anterior ramus. **E** Cirrus II. **F** Dorsal side of posterior ramus. **G**, **H** Bidentate serrate setae at posterior ramus. Scale bars in μm.

**Figure 6.**
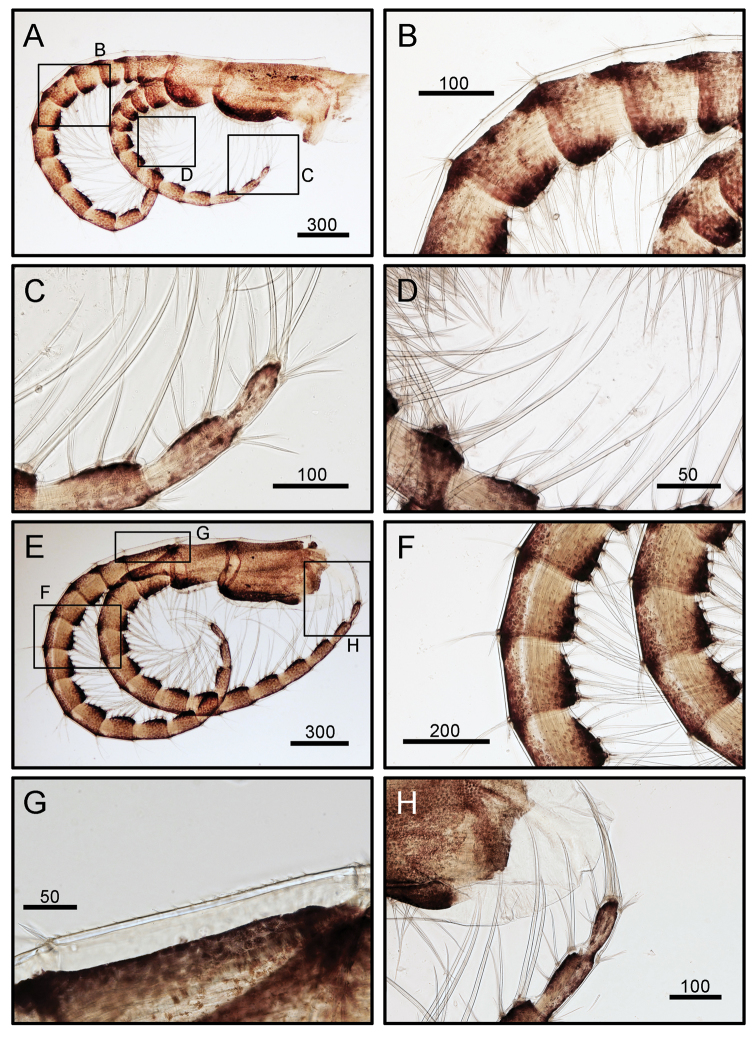
*Octomeris brunnea* (CEL-KT-131, Hai Kou, Taiwan). **A** Cirrus III. **B** Intermediate segments of posterior ramus of cirrus III. **C** Distal segments of anterior ramus of cirrus III. **D**. Simple type setae on anterior ramus of cirrus III. **E** Cirrus IV. **F** Intermediate segments of cirrus IV. **G** Dorsal surface of proximal segment of posterior ramus of cirrus IV. **H** Distal segments of anterior ramus of cirrus IV. Scale bars in μm.

**Figure 7.**
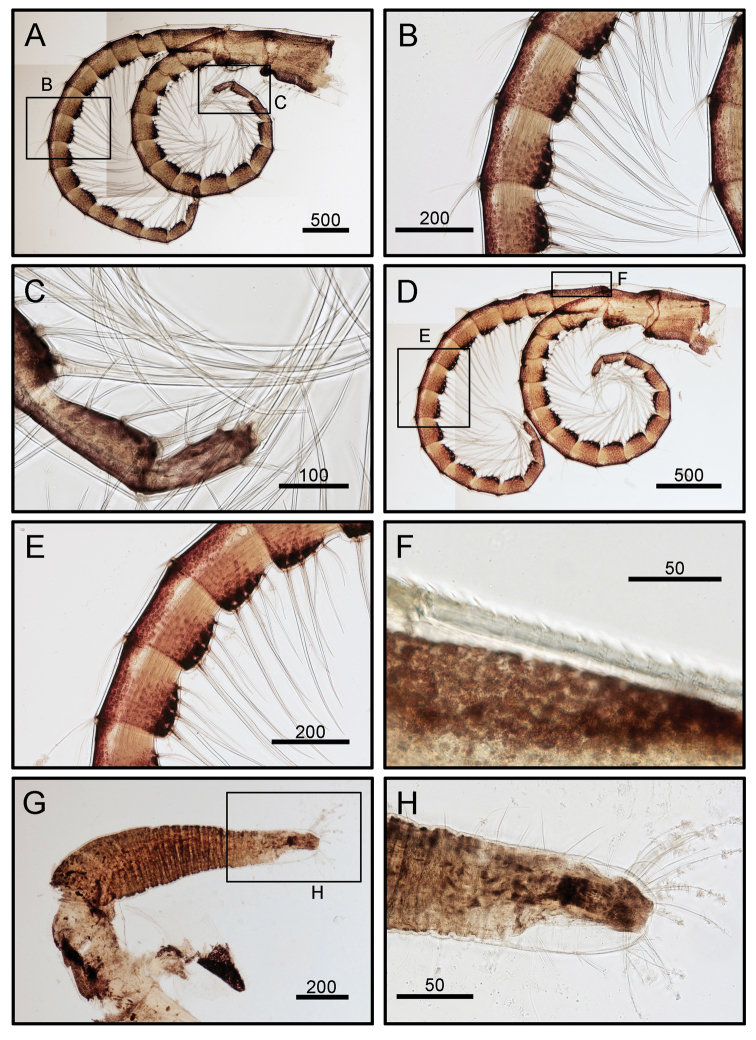
*Octomeris brunnea* (CEL-KT-131, Hai Kou, Taiwan). **A** Cirrus V. **B** Intermediate segments of posterior ramus of cirrus V. **C** Distal segments of anterior ramus of cirrus V. **D** Cirrus VI. **E** Intermediate segments of posterior ramus of cirrus VI. **F** Dorsal surface of proximal segments of posterior ramus of cirrus VI. **G** Penis. **H** Distal end of penis. Scale bars in μm.

Cirrus I rami unequal (Fig. 5A). Posterior ramus short, six-segmented. Anterior ramus seven-segmented. All segments height greater than width (Fig. 5A). Bidentate serrate setae and simple setae present. Bidentate serrate setae appear up to seven segments in anterior ramus and present up to first three distal segment in posterior ramus (Fig. 5B–D). Cirrus II, posterior ramus seven-segmented, anterior ramus eight-segmented. Bidentate serrate setae present up to seven segments in anterior ramus and up to first four distal segments in posterior ramus (Fig. 5E–H). Cirri III to VI similar in morphology, long and slender (Figs 6, 7). Cirrus III, posterior and anterior rami 12 segmented (Fig. 6A–D). Cirri IV and V, posterior and anterior rami 15-segmented (Figs 6E–H, 7A–C). Dorsal surface of cirri IV- VI has small spines (Figs 6G, 7F). Cirrus VI with 16 segmented rami (Fig. 7D–E). Intermediate segments of cirri III and VI with three pairs of long and one pair of short simple setae (Figs 6B, F, 7B, E). Distal segments of cirrus III bear two pairs of long and one pair of short setae (Figs 6C, D, H, 7C). Caudal appendage absent. Penis short, tip with a few simple setae (Fig. 7G, H).

**Figure 8.**
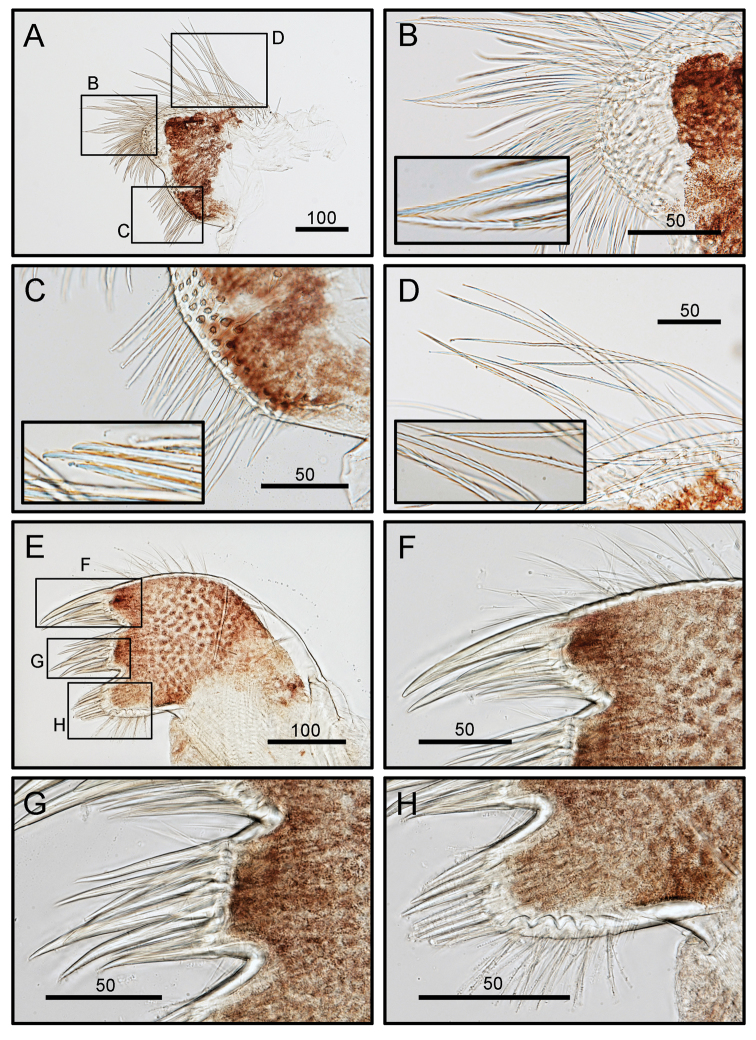
*Octomeris brunnea* (CEL-KT-131, Hai Kou, Taiwan). **A** Maxilla. **B** Magnified view of distal lobe showing serrulate setae. **C** Inner margin of proximal lobe of maxilla showing serrulate setae. **D** Outer margin of maxilla showing serrulate setae. **E** Maxillule; note the two deep notches on upper and lower 1/3 of the cutting edge. **F** Cutting edge above upper notch. **G** Middle portion of cutting edge. **H** Lower portion of cutting edge below lower notch. Scale bars in μm.

**Figure 9.**
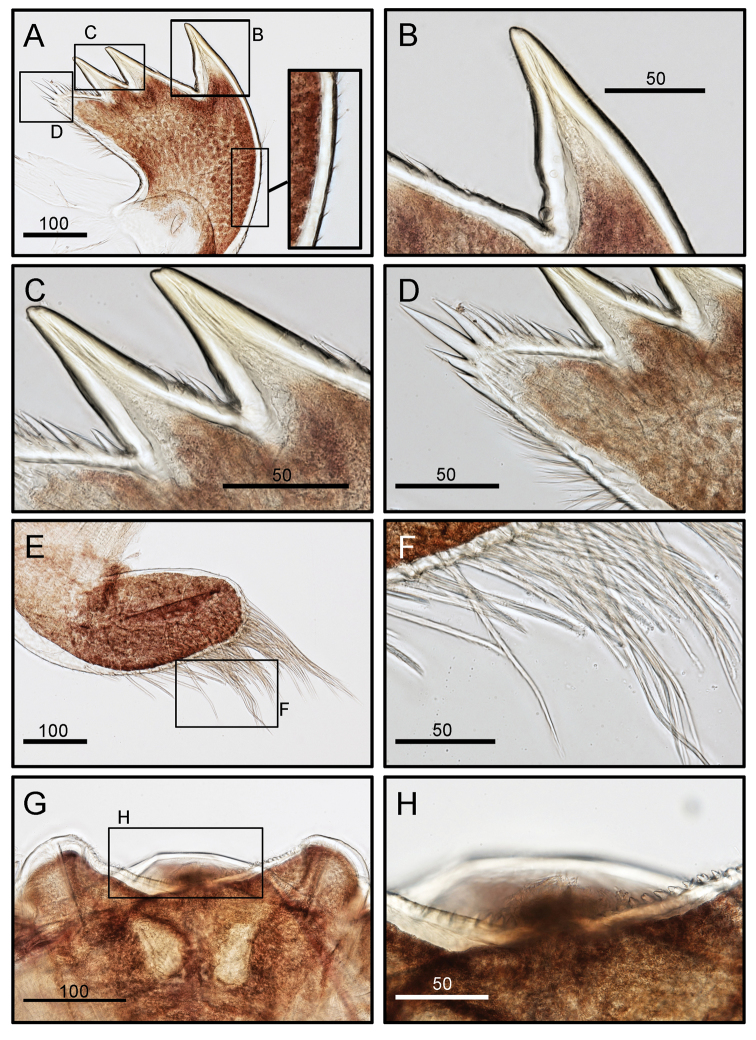
*Octomeris brunnea*. **A** Mandible. **B** First tooth of mandible. **C** Second and third teeth of mandible. **D** Lower margin. **E** Mandibular palp. **F** Serrulate setae at outer margin of mandibular palp. **G** Labrum. **H** Small teeth on middle part of cutting edge of labrum. Scale bars in μm.

Maxilla subtriangular, distal lobe prominent and proximal lobe flat, shallow notch present in inner margin between the two lobes (Fig. 8A), inner and outer margin with serrulate setae (Fig. 8B–D). Maxillule with a deep notch on upper 1/3 and lower 1/3 of cutting edge. Cutting edge obviously divided into three distinct portions. Cutting edge above upper notch with two large and a few setae (length of setae ranges from 80–100 μm); middle portion of cutting edge has six setae; 1/3 of lower portion of cutting edge has eight short setae (Fig. 8E–H). Mandibles with three teeth, cutting edge of first tooth smooth, second tooth with one or two spines, third tooth with a few spines on cutting edge (Fig. 9A–D). Mandibular palp elongated, with serrulate setae on outer margin (Fig. 9E, F). Cutting margin of labrum concave, with small fine teeth (Fig. 9G, H).

##### Distribution.

Southern Japan, Taiwan, Philippines, east coast of Queensland in Australia, Santa Cruz and New Hebrides (based on specimens in Australian Museum stated in Pope, 1965).

#### Octomeris intermedia

Taxon classificationAnimaliaSessiliaChthamalidae

Nilsson-Cantell, 1921

01BB691E-448B-5854-8761-EEC0A01DB5BA

Figures 2A–F , 10 , 11 , 12 , 13 , 14 , 15A–C , 16A–C

Octomeris brunnea Nilsson-Cantell, 1921: 303, figs 60, 61, pl 3, fig. 8.−1925: 1 (erratum for type locality); 1932: 13; 1938: 33, fig. 5; Hiro 1939: 252; Pope 1965: 21; Jones 2012: tabs 1, 2.

##### Material examined.

ASIZCR-000431. Intertidal rocks at Ao Nang Beach, Krabi, Thailand (8°02.06'N, 98°48.58E, 3 July 2019, 1 specimen). CEL-Thai-359. Intertidal rocks at Ao Nang Beach, Krabi, Thailand (8°02.06'N, 98°48.58'E, 3 July 2019, 20 specimens). CEL-Thai-243 Intertidal rocks at Hey Island, Phuket, Thailand (7°44.73'N, 98°22.59E, 15 May 2019, 103 specimens).

##### Diagnosis.

Shell eight plated, very depressed, surface brown with longitudinal furrows on uneroded specimens, tergo-scutal junction sinuous, except for young specimens. Maxillule with very shallow notch at upper 1/3 of cutting edge, lower 1/3 slightly protruded.

##### Description.

Shell eight-plated, composed of single rostrum (R) and carina (C), and paired rostro-laterals (RL), carino-laterals (CL) and laterals (L) (Fig. 2D–F). Shell very depressed, brown surface with longitudinal furrows on uneroded specimens (Fig. 2D–F). Tergal-scutal junction sinuous, except for young specimen which has shell length < 10 mm (Fig. 3). Sutures of shell plates serrated (Fig. 4C, D). Scutum triangular, outer surface with horizontal growth lines. Inner surface of scutum brown, occluding margin straight, basal margin slightly convex, tergal margin sinuous with deep articular ridge. Adductor muscle scar shallow (Fig. 4C, D). Basal margin of tergum strongly bended in angle, scutal margin sinuous and with deep articular ridge, crests of depressor muscle crests distinct, muscle crests extended slightly out of the carinal margin of tergum (Fig. 4C, D).

**Figure 10.**
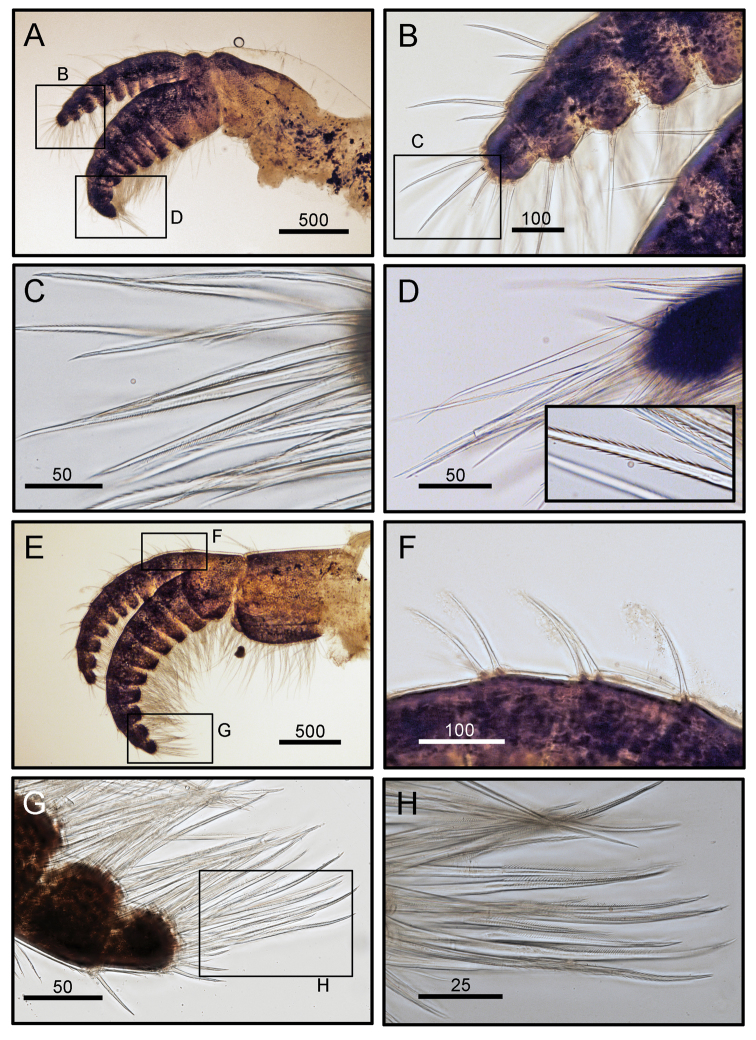
*Octomeris intermedia* (CEL-Thai-359, Krabi, Thailand). **A** Cirrus I. **B** Posterior ramus of cirrus I. **C** Bidentate serrate setae at tip of distal segment of posterior ramus. **D** Bidentate serrate setae at tip of anterior ramus. **E** Cirrus II. **F** Dorsal side of posterior ramus. **G**, **H** Bidentate serrate setae at posterior ramus. Scale bars in μm.

**Figure 11.**
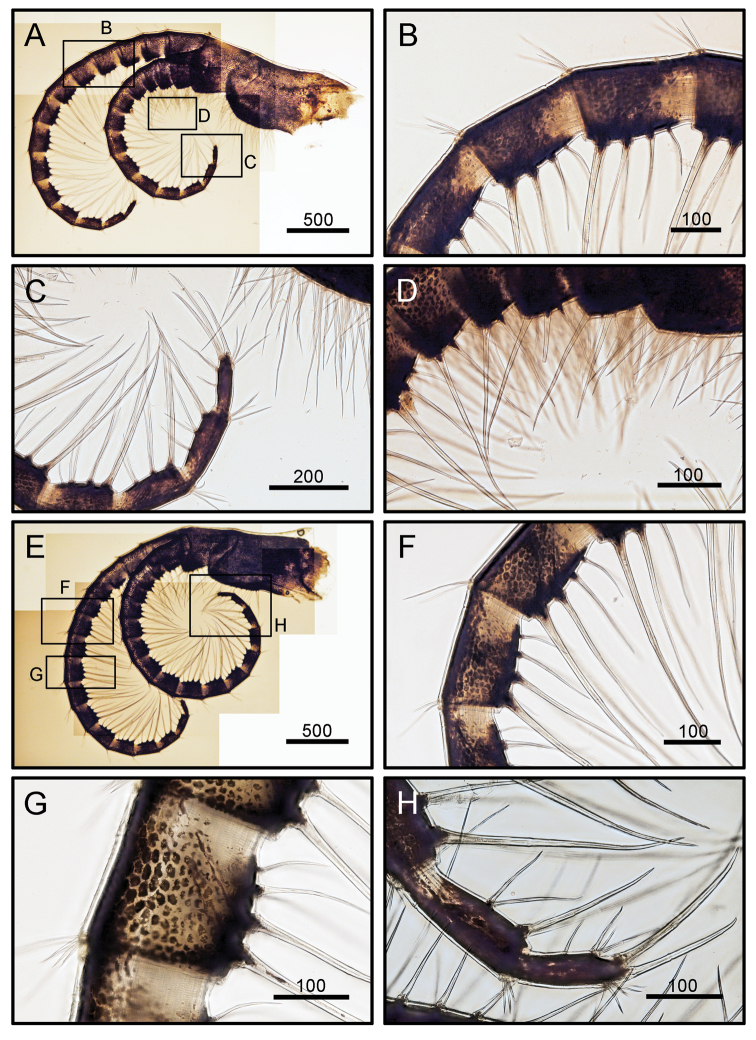
*Octomeris intermedia* (CEL-Thai-359, Krabi, Thailand). **A** Cirrus III. **B** Intermediate segments of posterior ramus of cirrus III. **C** Distal segment of anterior ramus of cirrus III. **D** Simple setae at intermediate segments of anterior ramus of cirrus III. **E** Cirrus IV. **F** Intermediate segments of posterior ramus cirrus IV. **G** Intermediate segment of posterior ramus of cirrus IV. **H** distal segment of anterior ramus of cirrus IV. Scale bars in μm.

**Figure 12.**
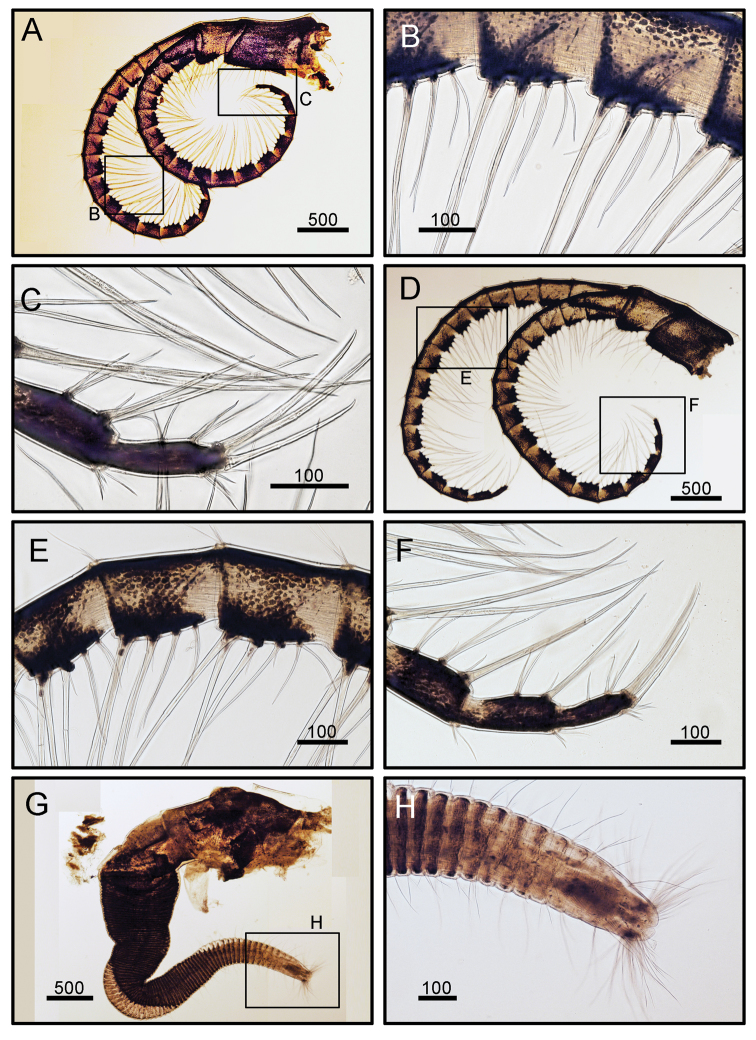
*Octomeris intermedia* (CEL-Thai-359, Krabi, Thailand). **A** Cirrus V. **B** Intermediate segments of posterior ramus of cirrus V. **C** Distal segments of anterior ramus of cirrus V. **D** Cirrus VI. **E** Intermediate segments of posterior ramus of cirrus VI. **F** Distal segment of anterior ramus of cirrus VI. **G** Penis. **H** Distal end of penis. Scale bars in μm.

Cirrus I, rami subequal (Fig. 10A–D). Posterior ramus shorter, eight-segmented. Anterior ramus seven-segmented. Segments in both rami with greater height than width. Both rami bear bidentate serrate setae and simple setae (Fig. 10C, D). Bidentate serrate setae present up to seven segments in anterior ramus and present up to first four distal segments in posterior ramus. Cirrus II, posterior ramus nine-segmented, anterior ramus ten-segmented (Fig. 10E, H). Bidentate serrate setae present up to six segments in anterior ramus and nine distal segments in posterior ramus (Fig. 10G, H). Cirri III–VI similar in morphology, being long and slender (Figs 11, 12). Cirrus III, anterior and posterior rami 12-segmented (Fig. 11A–D). Cirrus IV, anterior and posterior rami 16-segmented (Fig. 11E–H). Cirrus V, anterior and posterior rami 17 segmented (Fig. 12A–C). Cirrus VI, anterior ramus 17 segmented and posterior ramus 16 segmented (Fig. 12D–F). Intermediate segments of cirri III–VI bear three pairs of long and one pair of short simple setae (Figs 11B, D, F, 12B, E). Distal segments of cirrus III bear two pairs of long and one pair of short setae (Fig. 11C, H; 12C, F). Caudal appendages absent. Penis long, annulated, tip with simple setae (Fig. 12G, H).

**Figure 13.**
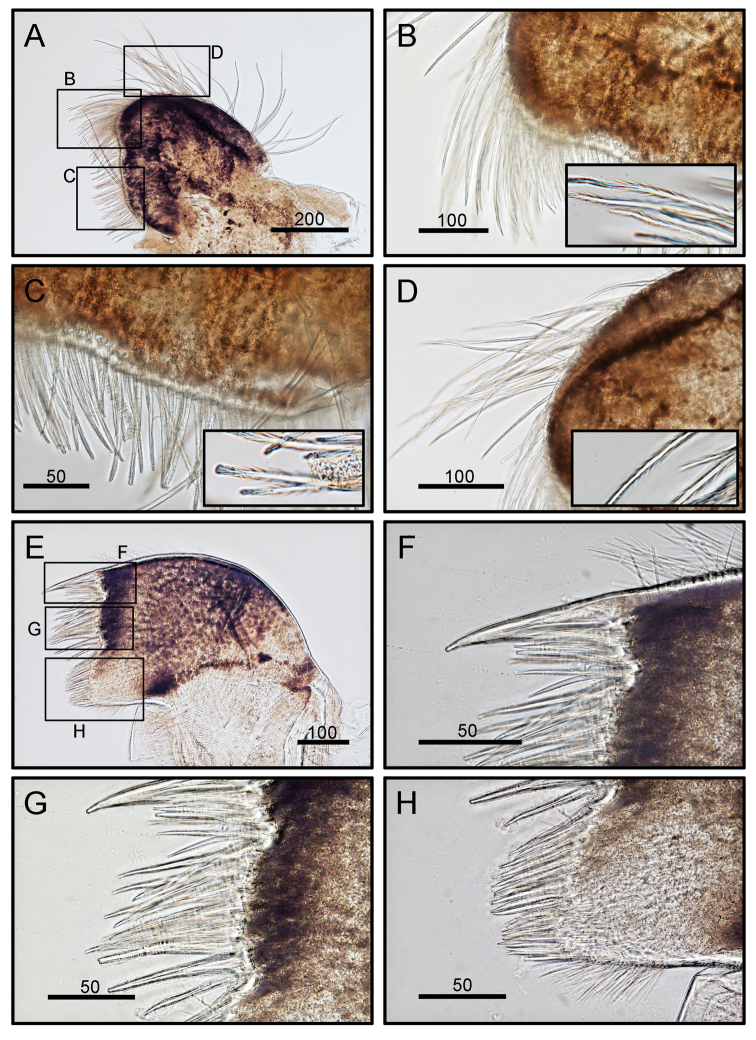
*Octomeris intermedia* (CEL-Thai-359, Krabi, Thailand). **A** Maxilla. **B** Magnified view of distal lobe showing serrulate setae. **C** Inner margin of maxilla showing serrulate setae. **D** Outer margin of maxilla showing serrulate setae. **E** Maxillule; note the two shallow notches on upper and lower 1/3 of the cutting edge. **F** Cutting edge above upper notch. **G** Middle portion of cutting edge. **H** Lower portion of cutting edge. Scale bars in μm.

**Figure 14.**
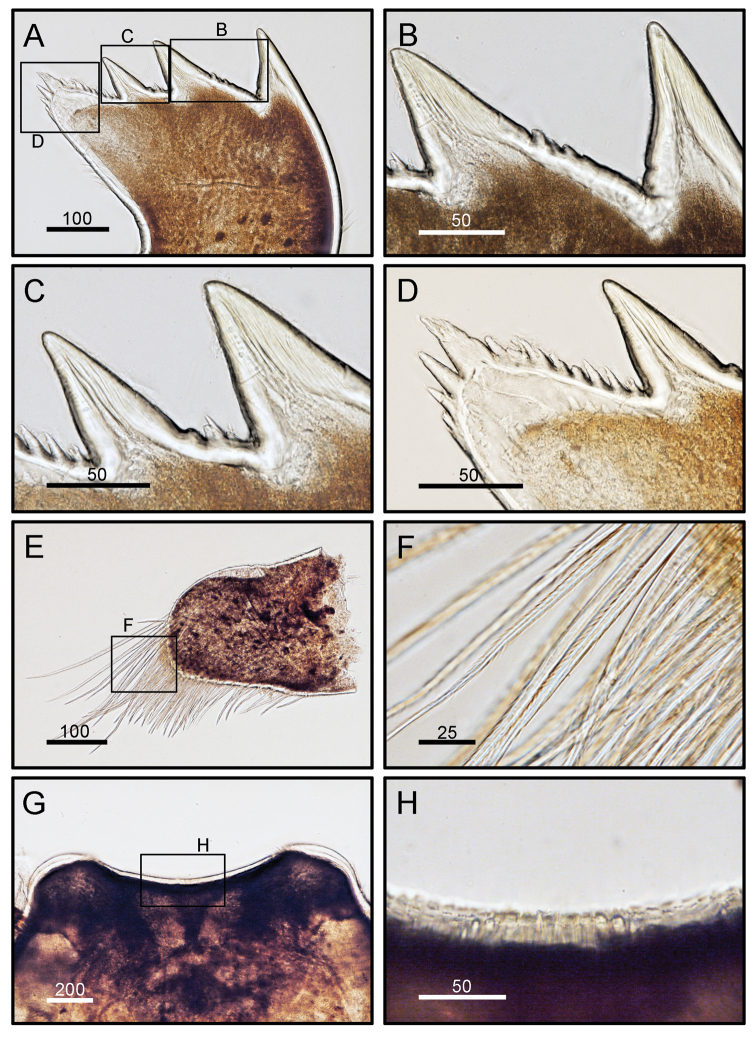
*Octomeris intermedia* (CEL-Thai-359, Krabi, Thailand). **A** Mandible, whole view **B** First and second teeth of mandible. **C** Second and third teeth of mandible of another specimen. **D** Lower margin of mandible. **E** Distal part of mandibular palp. **F** Serrulate setae at distal margin of mandibular palp. **G** Labrum. **H** Cutting edge of labrum, middle part. Scale bars in μm.

**Figure 15.**
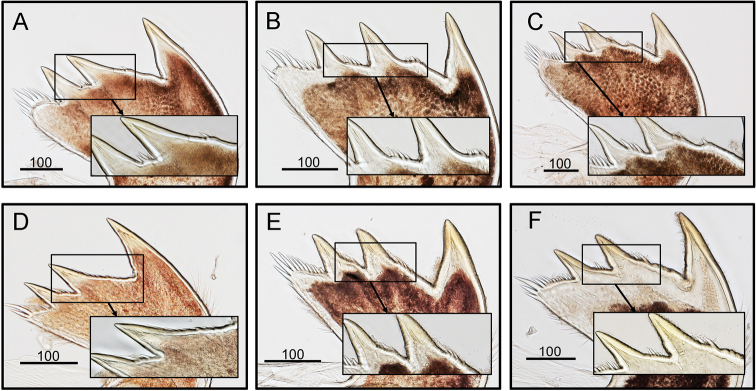
Variation in the occurrence of small spines along the cutting edge of second and third teeth of mandibles in *O. intermedia* (A-C) (CEL-Thai-359, Krabi, Thailand) and *O. brunnea* (D-F) (CEL-KT-131, Hai Kou, Taiwan). Scale bars in μm.

Maxilla subtriangular, inner margin with an inconspicuous notch, inner and outer margins with serrulate setae (Fig. 13A–D). Maxillule with two very shallow notches on upper 1/3 and lower 1/3 of cutting edge. Cutting edge more or less straight but the region above notch with one large and a few setae; middle margin has nine setae; cutting edge below upper notch has > 20 short setae (Fig. 13E–H). Mandibles with three teeth, first tooth with smooth edge and second teeth with slightly serrated edge, third tooth with smooth edge but occasionally with some spine on edge region (Figs 14A–D, 15A–C). Mandibular palp elongated, with serrulate setae on outer margin (Fig. 14E, F). Cutting margin of labrum concave, with small fine teeth (Fig. 14G–H).

##### Distribution.

Java in Indonesia, Mergui Archipelago in Myanmar, Phuket and Krabi in Thailand.

##### Remarks.

**Figure 16.**
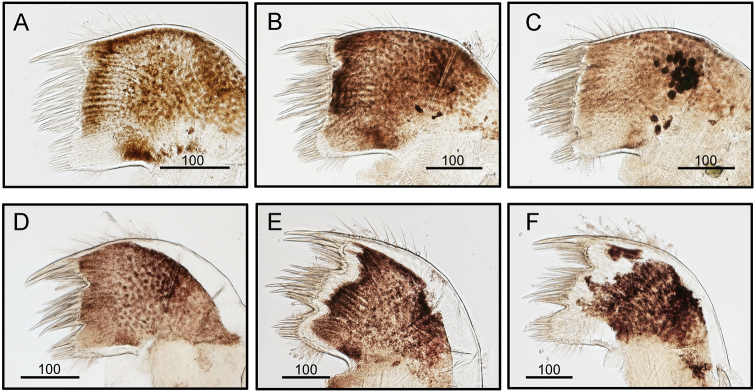
Consistent differences in the presences of shallow and deep notches on the cutting edge of *O. intermedia* (A-C) CEL-Thai-359, Krabi, Thailand) and *O. brunnea* (D-F) (CEL-KT-131, Hai Kou, Taiwan), respectively. The notches in *O. brunnea* are much deeper, and three distinct regions can be seen along the cutting edge. Scale bars in μm.

*O. intermedia* collected in the present study represents the specimens described by Nilsson-Cantell (1921, 1938) who collected *O. intermedia* from Java and the Mergui Archipelago; Phuket and Krabi are approximately 300 km south of the Mergui Archipelago. The morphology of our specimens fits the description in Nilsson-Cantell (1921, 1938): the shell is depressed and has a sinuous junction between the tergum and scutum. The external shell morphology of *O. brunnea* and *O. intermedia* is very similar. Old and eroded specimens of *O. brunnea* have a sinuous junction line between scutum and tergum, which is one of the characteristics of *O. intermedia* described by Nilsson-Cantell (1921). There are, however, several consistent diagnostic features between *O. intermedia* and *O. brunnea*. The shell of *O. intermedia* is much depressed in comparison to that of *O. brunnea*. The junction of the tergum and scutum in *O. intermedia* is sinuous, even at the young stage (except for very small individuals, RC-diameter < 10 mm; Fig. 3). In contrast, young individuals of *O. brunnea* have a straight junction between the scutum and tergum, and this junction line becomes sinuous when the barnacles get older (Fig. 3). There are some variations in the number of spines (–2–4) on the region between the second and third teeth of mandibles in *O. brunnea* and *O. intermedia* (Fig. 15). But both species have similar range of variations and there are no diagnostic differences between the two species (Fig. 15). The maxillule of *O. brunnea* has very deep notches on the upper and lower 1/3 portions of the cutting edge dividing it into three distinct portions. While the maxillule of *O. intermedia* has shallow notches on the upper and lower 1/3 portions of the cutting margin looking more or less straight without being dividing into three distinct regions as in *O. brunnea*. Such differences are considered consistent based on observations of the additional three specimens from both species (Fig. 16).

Nilsson-Cantell (1921) has not state any deposition nor specimen number of type or paratype specimens of *O. intermedia*. The foreword section of Nilsson-Cantell (1921) stated majority of specimens in Nilsson-Cantell (1921) were obtained from collections in Swedish Imperial Museum in Stockholm, Sweden and Zoological Museum in Uppsala, Sweden. The specimens of *O. intermedia* are possibly housed in either one of the two museums above. Before checking the presences or absences of *O. intermedia* in museum collections in Sweden, the present study did not attempt to establish any neotypes of *O. intermedia* to avoid taxonomic confusion. The information of the COI gene in GenBank for *O. intermedia* is currently adequate for future studies to confirm identification of specimens collected.

## Molecular analysis

**Figure 17.**
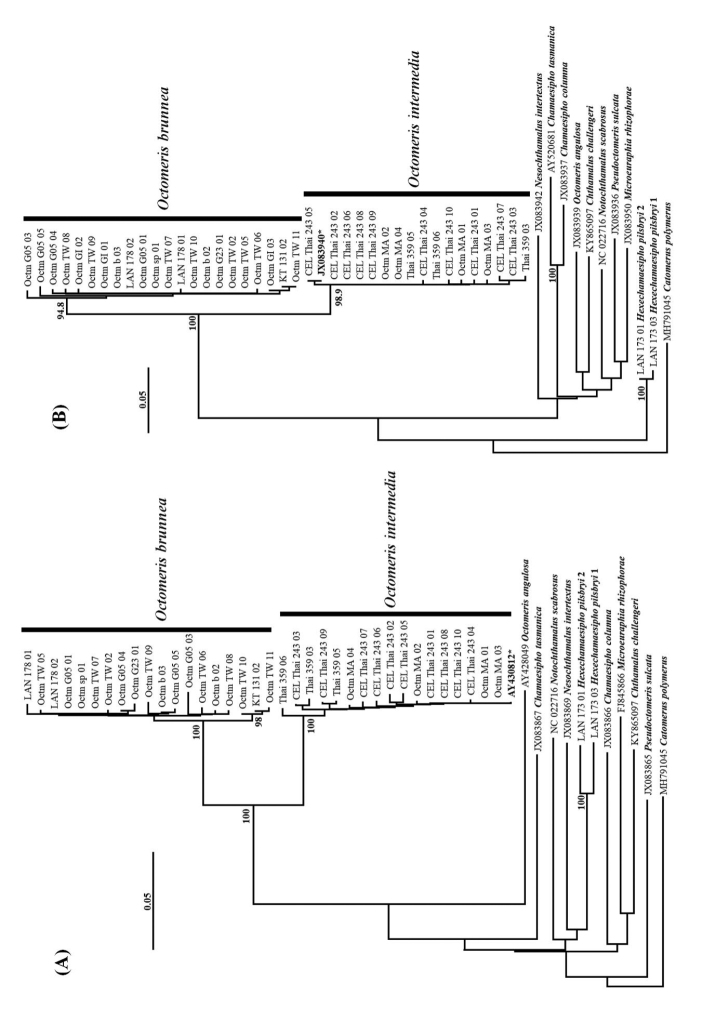
The neighbor-joining trees reconstructed with COI **A** and 12S rRNA **B** by MEGA X. Bootstrap values above 90 are represented at the nodes. “*” indicates the sequences from Fisher et al. (2004) and Pérez-Losada et al. (2012), which were designated as *O. brunnea* in their studies.

**Figure 18.**
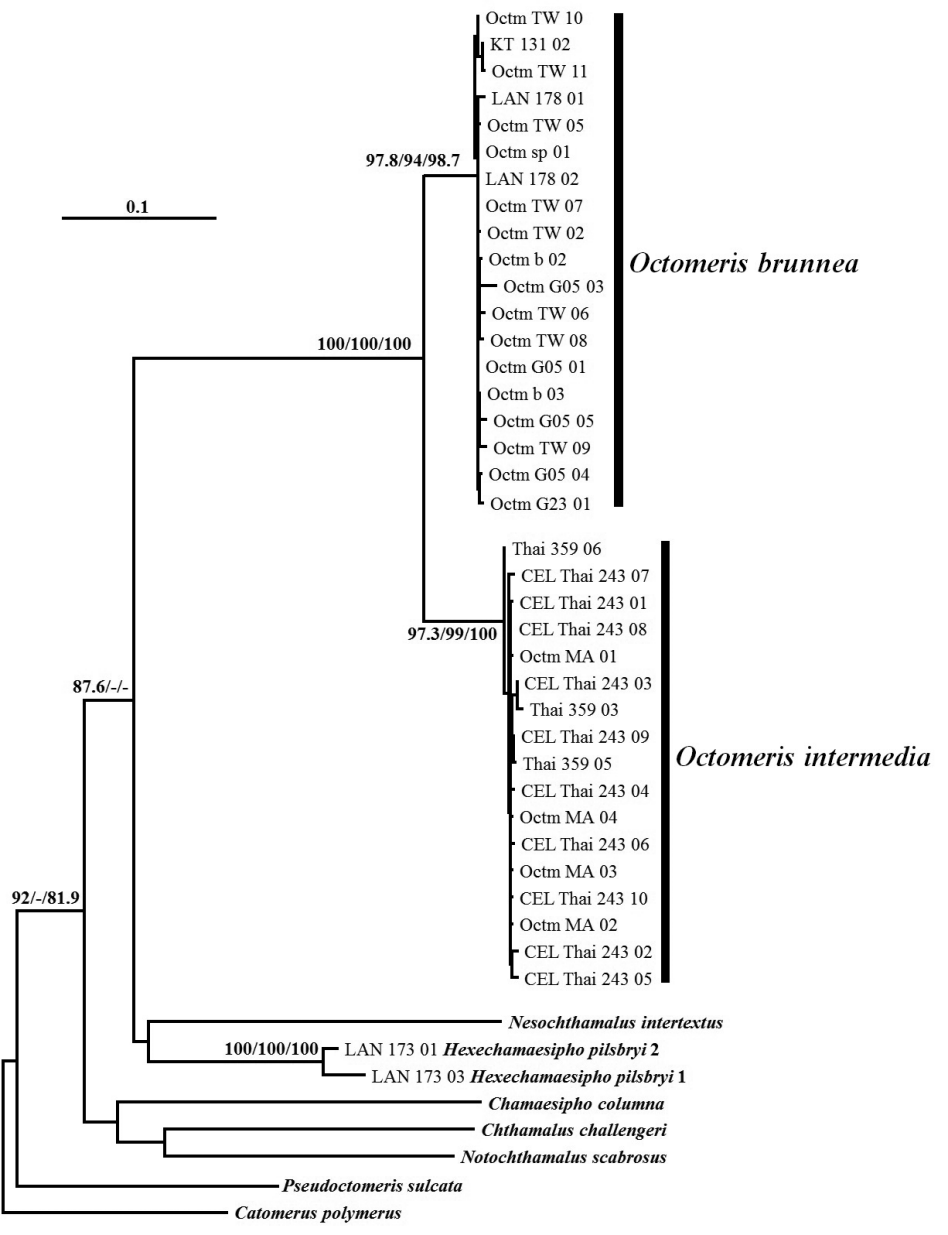
Maximum likelihood (ML) phylogenetic tree based on the COI and 12S rRNA sequences. The SH-aLRT support, ultrafast bootstrap support, and posterior probability (%) above 80 are represented at the nodes.

All the phylogenetic results suggested that both *Octomeris brunnea* and *O. intermedia* were clustered their own clades with high bootstrap values and posterior probabilities. The sequences from Fisher et al. (2004) and Pérez-Losada et al. (2012), which were designated as *O. brunnea*, were clustered with *O. intermedia* collected from Malaysia and Thailand by the NJ method (Fig. 17A, B). The phylogeny reconstructed by ML and BI suggested that *O. brunnea* and *O. intermedia* were sister groups (Fig. 18).

The K2P distances within *O. brunnea* and *O. intermedia* were 0.007±0.001 and 0.005±0.001 for the COI sequences, and 0.004±0.001 and 0.004±0.001 for the 12S rRNA sequences, respectively. The K2P distances between *O. brunnea* and *O. intermedia* were 0.098±0.013 and 0.043±0.001 for the COI and 12S rRNA sequences, respectively. The K2P distances between these two species and other species ranged from 0.207 to 0.251 for the COI sequences and 0.167 to 0.303 for the 12S rRNA sequences (Table 2).

**Table 2.** Kimura 2-parameter (K2P) distances of COI and 12S rRNA sequences between species by MEGA X. The lower left of the matrix are the mean distances, and the upper right of the matrix are the SD.

**(A) COI**												
	1	2	3	4	5	6	7	8	9	10	11	12
1. *Octomeris brunnea*		0.013	0.021	0.020	0.021	0.021	0.020	0.021	0.021	0.019	0.023	0.022
2. *O. intermedia*	0.098		0.020	0.020	0.020	0.019	0.022	0.021	0.020	0.020	0.021	0.023
3. *O. angulosa*	0.209	0.210		0.022	0.021	0.023	0.023	0.023	0.020	0.021	0.021	0.020
4. *Chamaesipho tasmanica*	0.208	0.215	0.243		0.019	0.019	0.022	0.021	0.020	0.018	0.020	0.022
5. *Pseudoctomeris sulcata*	0.226	0.231	0.212	0.193		0.019	0.020	0.022	0.020	0.018	0.020	0.020
6. *Chamaesipho columna*	0.232	0.218	0.247	0.204	0.207		0.019	0.020	0.020	0.018	0.020	0.021
7. *Nesochthamalus intertextus*	0.211	0.247	0.251	0.219	0.222	0.205		0.022	0.021	0.018	0.019	0.022
8. *Microeuraphia rhizophorae*	0.246	0.241	0.247	0.221	0.243	0.216	0.224		0.019	0.021	0.023	0.022
9. *Chthamalus challengeri*	0.237	0.220	0.199	0.196	0.202	0.200	0.220	0.186		0.019	0.019	0.021
10. *Hexechamaesipho pilsbryi*	0.201	0.207	0.199	0.164	0.175	0.179	0.167	0.217	0.195		0.019	0.019
11. *Notochthamalus scabrosus*	0.241	0.223	0.215	0.212	0.208	0.217	0.196	0.250	0.200	0.189		0.021
12. *Catomerus polymerus*	0.229	0.251	0.197	0.240	0.195	0.212	0.236	0.238	0.226	0.193	0.226	
**(B) 12S rRNA**												
	1	2	3	4	5	6	7	8	9	10	11	12.000
1. *Octomeris brunnea*		0.011	0.030	0.033	0.029	0.031	0.029	0.029	0.029	0.024	0.029	0.028
2. *O. intermedia*	0.043		0.028	0.036	0.030	0.032	0.030	0.028	0.029	0.024	0.028	0.029
3. *O. angulosa*	0.239	0.224		0.031	0.027	0.031	0.031	0.025	0.025	0.027	0.026	0.028
4. *Chamaesipho tasmanica*	0.279	0.303	0.260		0.034	0.018	0.036	0.033	0.036	0.031	0.033	0.032
5. *Pseudoctomeris sulcata*	0.239	0.248	0.213	0.285		0.030	0.029	0.025	0.031	0.024	0.026	0.027
6. *Chamaesipho columna*	0.263	0.275	0.258	0.115	0.246		0.034	0.030	0.033	0.029	0.031	0.029
7. *Nesochthamalus intertextus*	0.238	0.252	0.244	0.299	0.224	0.279		0.030	0.031	0.028	0.029	0.031
8. *Microeuraphia rhizophorae*	0.239	0.238	0.194	0.276	0.183	0.238	0.230		0.026	0.023	0.025	0.025
9. *Chthamalus challengeri*	0.223	0.231	0.182	0.301	0.264	0.283	0.245	0.193		0.028	0.028	0.027
10. *Hexechamaesipho pilsbryi*	0.167	0.170	0.207	0.252	0.179	0.239	0.220	0.159	0.210		0.027	0.021
11. *Notochthamalus scabrosus*	0.220	0.215	0.190	0. 281	0.200	0.248	0.228	0.177	0.213	0.204		0.026
12. *Catomerus polymerus*	0.211	0.219	0.225	0.273	0.206	0.243	0.265	0.200	0.219	0.145	0.200	

## Discussion

In the present study, we conclude that *Octomeris intermedia* is a valid species using integrative taxonomy. There are consistent morphological differences in the shell and maxillule of *O. intermedia* and *O. brunnea*, suggesting they are two distinct species. *Octomeris intermedia* is common in the west coast, on the Indian Ocean side of the Malay Peninsula. *Octomeris brunnea* is common in the Pacific Ocean and the South China Sea. Molecular analysis suggests that *O. brunnea* and *O. intermedia* are sister clades. However, *O. angulosa* collected by Pérez-Losada et al. (2012) is located outside the clades containing *O. brunnea* and *O. intermedia*. The close relationship between *O. intermedia* and *O. brunnea* in the phylogenetic analysis suggests that these two species may have formed when the Sunda Shelf was exposed during the Pleistocene glaciations, separating the Indian and Pacific Oceans (Voris 2000). Many sister taxa or distinct population genetic divergences in other marine species, including *Tetraclita* and *Chthamalus* barnacles and coral reef fishes, also formed when the Indian and Pacific Oceans separated during the last glacial maxima (Bowen et al. 2001; Tsang et al. 2011, 2012).

Fisher et al. (2004) and Pérez-Losada et al. (2012) included *Octomeris brunnea* in their phylogenetic studies. These *O. brunnea* were collected in Phuket, Thailand. In the phylogenetic analysis in the present study, the sequences of *O. brunnea* of Fisher et al. (2004) and Pérez-Losada et al. (2012) were clustered in the same clade as the *O. intermedia* collected from Phuket and Krabi in the present study, suggesting that these specimens of *O. brunnea* in Fisher et al. (2004) and Pérez-Losada et al. (2012) are *O. intermedia*. *Octomeris angulosa* is recorded from South African waters, and there are no other records outside this region. Fisher et al. (2004) included *Octomeris angulosa* from South Africa (region around the type locality) in their phylogenetic analysis, and the COI gene of this *O. angulosa* is a sister molecular clade with *O. brunnea* and *O. intermedia* in the present study (there are no 12S genes of *O. angulosa* in Fisher et al. 2004). Pérez-Losada et al. (2012) included *Octomeris angulosa* collected in Sydney, Australia in their phylogenetic analysis. According to Pope (1965) and Jones (2012), only *Octomeris brunnea* has been recorded in Australian waters. Only the 12S rRNA sequences of this *O. angulosa* from Pérez-Losada et al. (2004) was available for our analysis. The 12S rRNA sequence of *O. angulosa* from Pérez-Losada et al. (2012) located the taxon outside the clade containing both *O. intermedia* and *O. brunnea* in the present study. Future studies should focus on the diversity and taxonomy of *Octomeris* in Australia.

Intertidal barnacle diversity in Thailand received very little attention until the recent studies of Pochai et al. (2017) and Sukparangsi et al. (2019), who conducted detailed surveys of Thai intertidal barnacles and recorded a total of eleven species from the Thai coastline. The distribution of intertidal barnacles is different between the coastline in the Gulf of Thailand and the Andaman Sea (Pochai et al. 2017): the Andaman side has a higher species diversity (nine total species in Andaman side and six in the Gulf of Thailand). *Octomeris* was not reported by Pochai et al. (2017) or Sukparangsi et al. (2019). The record of *O. intermedia* in the present study brings the number of Thai intertidal barnacle species to 12. No *Octomeris* were found during sampling trips by the first author to Si-Chang Island and Chumporn in the Gulf of Thailand. This suggests that the coastlines in Thai waters that *O. intermedia* is located is probably the Andaman Sea. Therefore, there are ten species of intertidal barnacles on the Andaman side, and six in the Gulf of Thailand.

## Supplementary Material

XML Treatment for Octomeris brunnea

XML Treatment for Octomeris intermedia
